# Prevalence and risk factors of erectile dysfunction in cirrhotic patients: An observational study

**DOI:** 10.1080/2090598X.2023.2238933

**Published:** 2023-07-24

**Authors:** Mohamed Fakhry, Hamdy Mahfouz, Khalid Abdelazeem, Mohamed AbdelSabour, Nour Shaheen, Ahmed Fathy, Amro M. Hassan, Hazem Dief, Mohamed El-Nady, Mustafa A. Haridy, Omran Mohamed, Safwat Salama, Emad Abdelrazzak, Walid Saber, Tarek Mohamed, Maha Mohamed, Wael Esmat, Eman Fathy, Muhamed Abdelrahim, Rasha Maree

**Affiliations:** a Hepatology, Gastroenterology and Infectious Diseases Department, Faculty of Medicine, Al-Azhar University, Assiut, Egypt; b Dermatology, Andrology and Venereal Diseases Department, Faculty of Medicine Al-Azhar University, Assiut, Egypt; c Faculty of Medicine, Alexandria University, Alexandria, Egypt; d Urology Department, Faculty of Medicine, Al-Azhar University, Assiut, Egypt; e Internal Medicine, Kasr El-Einy School of Medicine, Cairo University, Cairo, Egypt; f Internal Medicine, Faculty of Medicine, Al-Azhar University, Assiut, Egypt; g Radiodiagnosis Department, Faculty of Medicine, Al-Azhar University, Assiut, Egypt; h Department of Tropical Medicine and Gastroenterology, Sohag University, Assiut, Egypt; i Clinical Pathology Department, Faculty of Medicine, Al-Azhar University, Assiut, Egypt; j Department of Dermatology, Venerology and Andrology, Faculty of Medicine, Assiut University, Assiut, Egypt; k Department of Tropical Medicine and Gastroenterology, Assiut University, Assiut, Egypt

**Keywords:** Erectile dysfunction, liver cirrhosis, HCV

## Abstract

**Background:**

Erectile dysfunction (ED) is a prevalent complication observed in male patients with liver cirrhosis; however, there is limited understanding of the etiological determinants responsible for its occurrence. The objective of this investigation is to explore potential contributory factors that underlie the development of ED in male patients with liver cirrhosis.

**Method:**

A cross-sectional study was conducted on 200 male patients with liver cirrhosis, who were divided into three groups according to the Child score. ED was studied using the International Index of Erectile Function (IIEF-5) Questionnaire and penile Doppler.

**Results:**

The prevalence of ED among the cirrhotic patients was 80%, and it was more frequent in patients with advanced liver disease (Child C). Penile venous leakage was observed in 20% of cirrhotic patients, which increased to 28.6% in those with advanced liver cirrhosis. Multivariate logistic regression analysis showed that age, low albumin levels, elevated INR, high hemoglobin levels, and Child C were predictors of ED in cirrhotic patients.

**Conclusion:**

Several clinical variables have been identified as potential contributors to the development of erectile dysfunction (ED) in patients with cirrhosis. These variables include advanced age, decreased levels of albumin, elevated INR, increased hemoglobin levels, and Child C classification. Early identification and treatment of these factors could potentially improve the quality of life for cirrhotic patients with ED. Notably, patients with ED in this population were observed to have elevated levels of INR, serum bilirubin, and hemoglobin, as well as reduced levels of serum albumin.

## Introduction

Erectile dysfunction (ED) is a significant concern for men, which refers to the persistent inability to achieve or maintain an adequate erection for satisfactory sexual activity. The prevalence of ED worldwide rises with age and ranges from 1% to 10% in men under 40 years old, while for men over 70, it can be as high as 50% to 100% [1,2].

The primary cause of penile erection is a response by the nervous and circulatory systems to neurotransmitters, including nitric oxide (NO). These neurotransmitters induce dilation of the cavernous arterioles and smooth muscle cells in the penis, resulting in increased blood flow [3].

Erectile dysfunction is a physical condition that patients experience subjectively, in addition to objective tests, questionnaires are often used to diagnose ED. Sexual disorders and dysfunction have multiple facets [4]. Several tools have been created to evaluate a variety of aspects related to sexuality, such as sexual knowledge, different types of sexual dysfunction, sexual desire, arousal, ejaculation, satisfaction, quality of life, and erection. Examples of frequently utilized measurement instruments include the Arizona Sexual Experience Scale (ASEX), the Sexual Functioning Questionnaire (SFQ), and the Changes in Sexual Functioning Questionnaire (CSFQ) [5–7]. The International Index of Erectile Function (IIEF) questionnaire is the most commonly employed diagnostic approach, which includes 15 questions to assess five distinct domains associated with sexual function [6].

Liver cirrhosis is a condition where the liver has undergone significant damage, resulting in a distorted architecture and the formation of nodules. This condition is typically irreversible during its advanced stages, and the only viable treatment option may be a liver transplant [8]. Patients with liver cirrhosis are vulnerable to several complications, which can considerably reduce their life expectancy [9].

ED is a prevalent issue in males with liver cirrhosis, with prevalence ranging from 25% to 92% [10]. Some of the comorbidities and risk factors associated with ED in males with liver cirrhosis include alcohol use, hypertension, diabetes, metabolic syndrome, and depression. Other factors like changes in sex hormones, malnutrition, and the use of drugs such as diuretics and nonselective beta-blockers can also be linked to ED [11]. ED can have a negative impact on health-related quality of life and can cause depression [12]. Measuring health-related quality of life is crucial in managing liver cirrhosis, and addressing specific symptoms like ED can enhance health-related quality of life in males with liver cirrhosis [13].

The study aimed to assess the possible predictors of ED in liver cirrhosis patients, including viral serology analysis and serum testosterone level. This article is important clinically because it provides evidence for the high prevalence and potential risk factors of erectile dysfunction (ED) in male patients with liver cirrhosis, a condition that can impair their quality of life and psychological well-being. The article adds to the current literature by conducting a cross-sectional study on a large sample of 200 cirrhotic patients with different degrees of liver disease severity, using a validated questionnaire and penile Doppler to assess ED. The article also performs a multivariate logistic regression analysis to identify the independent predictors of ED among various clinical and laboratory variables. The article also reveals some novel findings, such as the association between ED and low albumin levels, elevated INR, high hemoglobin levels, and penile venous leakage. These findings suggest that ED in cirrhotic patients may be related to impaired liver function, coagulation disorders, hypervolemia, and vascular abnormalities. These factors could be potential targets for intervention to improve ED and quality of life in cirrhotic patients.

## Methods

### Participates

This is a cross-sectional prospective study conducted on 200 male patients with liver cirrhosis at Hepatology, gastroenterology and infectious diseases department, the university hospital, from January 2022 to November 2022.

### Study design

The study included male patients with liver cirrhosis who were married and aged between 18 and 55 years old. Patients more than 55 years old or less than 18 years old were excluded as they are less likely to be sexually active. Patients who were treated with Interferon therapy in the last year were excluded as the medication may affect sexual function. Patients with renal failure, endocrine disorders, heart failure, neurological or psychosomatic disorders, hypertension, diabetes mellitus, or organic causes of ED were excluded to avoid confounding factors that may affect sexual function.

### Sample size

To determine the appropriate sample size for our study, we utilized the following formula: *N* = [(Za + Zbeta) ^2 * p1 * q1 + (Za + Zbeta) ^2 * p2 * q2]/(p1 - p2) ^2. Here, p1 and p2 represent the prevalence of erectile dysfunction (ED) in patients with different Child Turcot Pugh Scores, while q1 and q2 represent 1 - p1 and 1 - p2 respectively. Za refers to the Z value corresponding to the chosen confidence level, usually 95% (which is 1.96), while Zbeta refers to the Z value corresponding to the chosen power, 80% (which is 0.84) [14].

We calculated the sample size for an analytical cross-sectional study comparing the prevalence of erectile dysfunction (ED) between patients with different Child Turcot Pugh Scores. Based on a systematic review and meta-analysis, we assumed that the prevalence of ED was 53.6% in Child A patients, 70% in Child B patients, and 88.4% in Child C patients. We wanted to detect a difference of 15% between Child A and Child B patients, and a difference of 18.4% between Child B and Child C patients. We also assumed a confidence level of 95% and a power of 80%. Using the formula for sample size calculation, we found that we needed at least 125 patients in each group for the Child A vs Child B comparison, and at least 68 patients in each group for the Child B vs Child C comparison [15].

### Procedures

Clinical examination was performed, and routine laboratory investigations were conducted, including CBC, serum creatinine, urea, free Testosterone, ESR, and liver function tests. The patients were classified according to Child Turcot Pugh Score into Child A, Child B, and Child C. Viral serology analysis was performed to test for HBV and HCV. Serum testosterone level was measured. Erectile dysfunction was evaluated using the Arabic version of the International Index of Erectile Dysfunction (IIEF-5) questionnaire. Penile Doppler ultrasound was also performed to assess the patients’ erectile function [6].

Penile Doppler ultrasound was performed to evaluate the patients’ penile blood flow and detect any abnormalities.

The primary measure used in the study was the Arabic version of the International Index of Erectile Dysfunction (IIEF-5) questionnaire, which assesses five domains of male sexual function: erectile function, orgasmic function, sexual desire, intercourse satisfaction, and overall satisfaction. The IIEF-5 scores range from 1 to 25, and ED and its severity were defined based on the scores: 4 or lower (no attempts at intercourse), 5 to 7 (severe ED), 8 to 11 (moderate ED), 12 to 16 (mild to moderate ED), 17 to 21 (mild ED), and 22 to 25 (‘normal’ erectile function, i.e. absence of ED)) [16]. The secondary measure used in the study was penile Doppler ultrasound, which measured penile blood flow parameters, including PSV, EDV, and the RI.

Penile Doppler ultrasound was performed to assess penile blood flow. The penile Doppler ultrasound was performed with the patient supine in a quiet, darkened room by the same operator using a machine (SIEMENS) with a 10 MHz linear array probe. An initial real-time examination, in both the transverse and longitudinal planes, was performed to study cavernosal anatomy and identify fibrous plaques. A rubber band tourniquet is placed around the base of the penis before injection. About 50–120 mg papaverine was injected into either corpus cavernosum using a 27- or 30-gauge needle. The penis was inspected for abnormal curvature and the shaft was palpated for fibrous thickening. The Scanning is made from the base at the penoscrotal junction, with the examiner’s wrist resting on the patient’s pubis. The Doppler signals were recorded from the cavernosal artery over a non-tortuous segment close to the root of the penis and care was taken to ensure angle correction to 60° and sample gate 1 mm during sampling. The accurate location of the Doppler gate further ensured optimal waveform tracing. The following parameters were measured at peak response from the clearest waveform obtained: the peak systolic velocity (PSV), the end-diastolic velocity (EDV) and the Resistance index (RI), (RI = PSV-EDV\PSV). The velocity waveforms from both cavernosal arteries at the same points were recorded initially at 5 min. and 10 min. 15 min. and 20 min post-injection. Avascular anatomy was also assessed for anomalies in flow and morphology. A (PSV) of less than 25 cm/sec was used as the threshold for arterial insufficiency. An (EDV) of greater than 5 cm/sec was used to predict venous incompetence. The images were recorded and printed on paper [17].

### Ethics consideration

The study was conducted under Helsinki standards as revised in 2013 and approved by the Ethics Committee of the University Faculty of Medicine. The patients were informed about the study’s purpose, and those who agreed to participate provided verbal and written informed consent. The patients’ privacy was guaranteed, and their data were kept confidential.

### Statistical analysis

The statistical analysis plan for this study involved the use of descriptive statistics and inferential statistics. Descriptive statistics were used to summarize the baseline characteristics and laboratory data of the studied patients, which included mean and standard deviation for continuous variables and frequency and percentage for categorical variables. Inferential statistics were used to investigate the prevalence of ED in cirrhotic patients, the relation of age groups and penile Doppler with ED, the frequency and severity of ED according to Child classification, the relation between ED and the cause of liver cirrhosis, and the factors associated with ED using multivariate logistic regression analysis. The Chi-square test was used to determine the statistical significance at *p* < 0.05. Confidence intervals were reported for the odds ratio estimates.

## Results

### Baseline characteristics of the included patients

The study enrolled 200 participants, with an average age 45.8 years (SD = 8.1 years). Among them, 140 participants (70%) reported smoking. Liver cirrhosis was predominantly caused by HCV (72.5%), followed by HBV (17.5%) and cryptogenic causes (10%). The Child score classified 45% of the patients as Child A, 20% as Child B, and 35% as Child C. The testosterone levels were normal in 65% of the patients, while 35% had below-normal levels. The demographic information for the patients under study is presented in Table 1.

**Table 1.** Baseline characteristics and laboratory data of the studied patient.

Age		
Mean± SD		45.8 ± 8.1
Range		27–55
**Smoking**		
Yes		140 (70%)
No		60 (30%)
**Cause of liver cirrhosis**		
HCV		145 (72.5%)
HBV		35 (17.5%)
Cryptogenic		20 (10%)
Child Score		
Child A		90 (45%)
Child B		40(20%)
Child C		70 (35%)
**Testosterone level**		352.3 ± 155.4 (ng/dL)
Normal		130 (65%)
Below than normal		70 (35%)
**laboratory data**		
**Test**	**Mean±SD**	**Median and Range**
Albumin	3.09±0.96	3.7 (1.3–5)
Total bilirubin	2.22±1.8	9.6 (0.4–10)
ALT	60.9±20.7	87 (18–105)
AST	64.9±28.6	144 (28–172)
Prothrombin time	13.7±1.9	15.9 (11.7–17.6)
INR	1.3±0.28	1.2 (1–2.2)
Urea	34.9±13	51 (15–66)
Create	0.82±0.26	1 (0.3.–1.3)
ESR	31.9±16	80 (5–85)
Glucose	101.2±17.1	62 (71–133)
Hemoglobin	12.3±1.8	7.8 (8.3–16.1)
Platelets	83.3±75.6	116 (39–155)
WBC	6.5±2.22	11.3 (2.5–13.8)

### Prevalence of ED in cirrhotic patients

Among the cohort of 200 patients, 160 (80%) were diagnosed with erectile dysfunction (ED) while the remaining 40 (20%) were not. Of those patients diagnosed with ED, 25 (12.5%) presented with mild ED, and an additional 25 (12.5%) presented with mild-to-moderate ED. Among the patients with ED, 45 (22.5%) were diagnosed with moderate ED, and 65 (32.5%) presented with severe ED Table 2.

**Table 2.** The prevalence of ED in cirrhotic patients.

Patients	Frequency
Without ED	40 (20%)
With ED	160 (80%)
Mild ED	25 (12.5%)
Mild to moderate ED	25 (12.5%)
Moderate ED	45 (22.5%)
Severe ED	65(32.5%)

### Incidence of erectile dysfunction (ED) according to age

Table 3 shows the relationship between age groups and ED. There also indicates that there is a statistically significant relationship between age group and ED,

**Table 3.** Relation of age groups and penile Doppler with ED; Age group study showed that the age group (44–55) had a significantly higher prevalence of ED.

Age groups	Events(N)	Normal patients (40)	Patients with ED (160)	P-value
18–30	20	15(75.0%)	5(25.0%)	<0.001 S
31–43	40	5(12.5%)	35(87.5%)	
44–55	140	20(14.3%)	120(85.7%)	
**Doppler**				
Normal penile Doppler	160	35(21.9%)	125(78.1%)	**>0.05 NS**
Venous leakage	40	5(12.5%)	35(87.5%)	

*Chi-square test was used.

**Statistically significant at *p* < 0.05.

The shows that younger patients (18–30) are less likely to have ED, with 75% of patients in this age group being normal and only 25% having ED. In contrast, the majority of patients in the older age groups (31–43 and 44–55) have ED, with 87.5% and 85.7% of patients in these groups having ED, respectively.

### Relation between penile Doppler results and ED

Table 3 shows the relation between penile Doppler results and ED. The table indicates that out of the 200 patients studied, 80% had normal penile Doppler results, while 20% had penile venous leakage. Among the patients with normal penile Doppler results, 21.9% were normal patients and 78.1% had ED. Among patients with penile venous leakage, 12.5% were normal patients, and 87.5% had ED. However, there is no statistically significant relationship between penile Doppler results and ED.

### Frequency and severity of ED according to Child Classification

Research findings demonstrate a significant association between age groups and ED, whereby younger patients aged 18–30 exhibit lower prevalence rates of ED. Specifically, 75% of patients in this age group exhibit normal sexual function while only 25% manifest ED. Conversely, a higher proportion of patients in older age categories, specifically those aged 31–43 and 44–55, exhibit ED with 87.5% and 85.7% of patients in these groups presenting with ED, respectively Table 4.

**Table 4.** Frequency and severity of ED according to Child Classification.

ED category	Child class A *N*=90	Child class B *N*=40	Child class C N= 70	P. Value
No ED (40)	35(38.9%)	0(0.0%)	5(7.1%)	<0.0001S
Mild ED (25)	25(27.8%)	0(0.0%)	0(0.0%)	
Mild to moderate ED (25)	5(5.6%)	15(37.5%)	5(7.1%)	
Moderate ED (45)	5(5.6%)	20(50.0%)	20(28.6%)	
Severe ED (65)	20(22.2%)	5(12.5%)	40(57.1%)	

*Chi-square test was used.

**Statistically significant at *p* < 0.05.

### Relation between ED and the cause of liver cirrhosis

incidence of erectile dysfunction (ED) was found to be higher in patients with liver cirrhosis related to Hepatitis C virus (HCV) (86.2% with ED) compared to those with liver cirrhosis related to Hepatitis B virus (HBV) (57.1% with ED) and cryptogenic liver cirrhosis (75% with ED) (P-value <0.001) Table 5.

**Table 5.** Relation between ED and the cause of liver cirrhosis.

Etiology of cirrhosis (Number)	Normal patients (40)	Patients with ED (160)	P-value
HBV-related (35)	15(42.9%)	20(57.1%)	**<0.001 S**
HCV-related (145)	20(13.8%)	125(86.2%)	
Cryptogenic (20)	5(25.0%)	15(75.0%)	

*Chi-square test was used.

**Statistically significant at *p* < 0.05.

### Multivariate logistic regression analysis

In the multivariate logistic regression analysis, several factors were found to be associated with ED. Age group 44–55 (*p* = 0.002), albumin <2.8 mg/dl (*p* < 0.001), Hb >16 g/dl (*p* < 0.001), Child class C (*p* = 0.02), and lower testosterone level (*p* = 0.027) were all significant predictors of ED Table 6.

**Table 6.** Multivariate logistic regression analysis of factors associated with ED.

			95% Confidence interval	
Parameters	P-value	Odd’s ratio	Lower	Upper
Age grop 31–43	0.998	0.52	0.65	1.57
Age group 44–55	**0.002**	1.023	0.02	0.9
Albumin 2.8–3.5 mg/dl	0.998	0.09	0.09	0.22
Albumin < 2.8 mg/dl	**<0.001**	1.42	1.21	1.32
Total bilirubin 2–3 mg/dl	0.997	0.21	2.36	3.36
Total bilirubin > 3 mg/dl	0.997	1.42	1.78	4.012
INR 1.7–2.2	**0.042**	1.12	0.13	0.34
Mild ascites	0.998	0.52	0.52	4.3
Moderate ascites	0.52	0.89	0.1	1.6
Marked ascites	0.998	0.03	0.39	1.36
Hb 12–16 g/dl	0.999	.000	2.56	5.06
Hb > 16 g/dl	**<0.001**	1.62	4.63	5.02
HCV-related cirrhosis	0.994	0.09	1.58	6.33
Cryptogenic cirrhosis	0.998	0.25	1.88	4.12
Child class B	0.999	0.85	5.12	8.36
Child class C	**0.02S**	1.12	6.14	9.15
Testosterone level	0.**027**	0.345	0.002	0.030

## Discussion

The findings of this study reveals that the prevalence of erectile dysfunction (ED) among cirrhotic patients was found to be increased than the prevalence reported in the general population. The prevalence of ED among the patients was found to be 80%, with the majority of patients having at least moderate ED. The study found a significant relationship between age and ED, with older patients being more likely to have ED. The severity of liver disease, as measured by the Child classification, was also found to be significantly associated with ED. Patients with more severe liver disease were more likely to have ED. The cause of liver cirrhosis was also found to be significantly associated with ED, with patients with HCV-related cirrhosis being at an increased risk of developing ED. The study’s finding that patients with HCV-related cirrhosis are at an increased risk of developing ED than those with HBV-related cirrhosis or cryptogenic cirrhosis is noteworthy.

Our research revealed that 80% of the patients we examined had ED, a finding that is consistent with Huyghe et al.‘s research from 2009, which reported a prevalence of 76% in candidates for liver transplantation with end-stage liver disease [18]. The high incidence of ED in cirrhotic patients could be attributed to various factors, such as decreased metabolic clearance rates, reduced testosterone response to human chorionic gonadotropin stimulation, increased estradiol and sex steroid binding globulin levels, as well as increased luteinizing hormone and follicle-stimulating hormone levels [19].

On the other hand, Jagdish et al.‘s study in 2022 reported a comparable result to our study, with 72.3% of men with cirrhosis experiencing ED [10]. The difference in reported ED prevalence is most likely due to variations in the severity of cirrhosis and differences in the assessment tools used. In contrast, Kim et al.‘s study reported a decreased prevalence of ED, with only 41.2% of participants experiencing it [20]. The patient selection criteria used in each study may account for this difference, as Kim et al.‘s patients were relatively young, stable, and at the compensated stage of their chronic disease without any hormonal or physical deterioration.

The study found that cirrhotic patients classified as Child C were more likely to report erectile dysfunction (ED) and experience more severe ED compared to those classified as Child A. Among Child A patients, 55 (61.1%) reported ED, with 20 (22.2%) experiencing severe ED. In contrast, 65 (92.9%) of Child C patients reported ED, with 40 (57.1%) experiencing severe ED, consistent with previous research by Huyghe et al. (2009) [18]. The increased prevalence and severity of ED in cirrhotic patients may be linked to hormonal changes, malnutrition, and drug use, including diuretics and nonselective beta-blockers [11].

Furthermore, the study used penile Doppler testing to assess blood flow to the penis and found that 160 (80.0%) patients had normal results while 40 (20.0%) had venous leakage. There was a statistically significant association between Child classification and penile Doppler results, with 70 (77.8%) of Child A patients having normal results and 20 (22.2%) having venous leakage. All 40 (100.0%) Child B patients had normal results, while 50 (71.4%) Child C patients had normal results and 20 (28.6%) had venous leakage (P-value <0.001).

The study also examined the relationship between the etiology of cirrhosis and ED. The results revealed that patients with liver cirrhosis due to HBV had an increased prevalence of ED (57.1%). This finding is consistent with previous research by Kim et al., whose study reported a prevalence of HBV-related liver cirrhosis of 36.7%. However, the sample size of Kim et al.‘s study was smaller than that of the current study [20].

The relationship between ED and the cause of liver cirrhosis was examined in this study, which found that patients with HCV-related liver cirrhosis had a significantly increased prevalence of ED (86.2%). This result is in agreement with Fábregas et al. [21], who also found a significant association between sexual dysfunction and liver cirrhosis, although the percentage was decresed (45.1%). One possible explanation for the difference in percentages is the patient selection criteria used in the evaluation of ED; Fábregas et al.‘s study included relatively young patients, while the current study had a larger sample size.

In addition, the study showed a positive correlation between age and ED, with the age group of 44–55 having a significantly higher prevalence of ED. This result is consistent with previous research by Maimone et al. [22], who found that patients with ED were significantly older than those without ED (p.value = 0.006). The morphologic and physiologic mechanisms involved in the aging process are thought to play a key role in the development of sexual dysfunction in the absence of any other clinical or medical condition. A meta-analysis by Yoo et al [15], also found that patients with ED were 5.8 years older (*p* < 0.001). However, Kim et al. concluded that age is not a risk factor for ED in cirrhotic patients, although their study had a smaller sample size [20].

As regards predictors of ED, multivariant logistic regression showed that Age group (44–55) years, Albumin less than 2.8 g/dl, INR range (1.7–2.2), Hemoglobin level more than 16 g/dl and Chid C can predict ED. The explanation of patients with Hb >16 g/dl having ED may be due to increased hemoglobin levels associated with sluggish blood flow. The study’s identification of low albumin levels, INR range 1.7–2.2, and Child class C as significant predictors of ED in cirrhotic patients has important clinical implications. These parameters can serve as screening tools for healthcare providers to identify cirrhotic patients at risk for ED. Healthcare providers can also use these parameters to monitor cirrhotic patients for the development of ED over time.

### Study limitations

The study employed a rigorous methodology to examine the prevalence of erectile dysfunction (ED) and its associated factors using validated tools such as the International Index of Erectile Dysfunction (IIEF-5) questionnaire and Penile Doppler ultrasound. However, it is difficult to completely avoid limitations in this type of clinical research. In this study, one limitation is the absence of intervention-based tests such as liver transplantation or interferon therapy. The study did not explore the potential impact of various treatments for cirrhosis, such as transjugular intrahepatic portosystemic shunt (TIPS) or liver transplantation, on ED. One more restriction of the research is that the patients were not monitored afterward. This highlights the need for additional future studies to establish a causal relationship between the risk factors identified in this study and erectile dysfunction in individuals with cirrhosis, thereby enhancing the evidence base.

## Conclusion

The study highlights the high prevalence of erectile dysfunction (ED) among cirrhotic patients, with 80% of patients experiencing ED. The study also found a significant association between age, severity of liver disease as measured by the Child classification, and the cause of cirrhosis with ED. Additionally, the study revealed a positive correlation between age and ED, with patients aged 44–55 having a significantly increased prevalence of ED. Penile Doppler testing showed that the majority of patients had normal results, supporting the conclusion that liver cirrhosis is the cause of ED in these patients. The findings of this study have important clinical implications for the management of cirrhotic patients, as ED can significantly impact their quality of life. Healthcare providers should consider screening for ED among cirrhotic patients and implementing appropriate interventions to improve sexual function. Future research should focus on identifying the mechanisms underlying the development of ED in cirrhotic patients and examining the effectiveness of various treatment modalities. This may lead to the development of more targeted interventions to improve sexual function in cirrhotic patients and enhance their overall quality of life.

## Data Availability

The datasets used and/or analyzed during the current study are available from the corresponding author upon reasonable request.
